# Rapid Nongenomic Action of Aldosterone on Protein Expressions of Hsp90(**α** and **β**) and pc-Src in Rat Kidney

**DOI:** 10.1155/2013/346480

**Published:** 2013-01-22

**Authors:** Somchit Eiam-Ong, Kittisak Sinphitukkul, Krissanapong Manotham, Somchai Eiam-Ong

**Affiliations:** ^1^Department of Physiology, Faculty of Medicine, Chulalongkorn University, Bangkok 10330, Thailand; ^2^Department of Medicine, Lerdsin General Hospital, Bangkok 10500, Thailand; ^3^Division of Nephrology, Department of Medicine, Faculty of Medicine, Chulalongkorn University, Bangkok 10330, Thailand

## Abstract

Previous *in vitro* studies indicated that aldosterone nongenomically phosphorylates epidermal growth factor receptor (EGFR) through activation of upstream signals, heat shock protein 90**β** (Hsp90**β**), and cytosolic (c)-Src kinase. We demonstrated that aldosterone rapidly elevates EGFR phosphorylation in rat kidney. There are no *in vivo* data regarding renal Hsp90(**α** and **β**) and phosphorylated (p)c-Src protein expressions. The present study further investigates the expressions of these proteins. Male Wistar rats were intraperitoneally injected with normal saline solution or aldosterone (Aldo: 150 **μ**g/kg BW). After 30 minutes, abundances and localizations of these proteins were determined. Aldosterone enhanced renal Hsp90**β** protein abundance (*P* < 0.001), but Hsp90**α** and pc-Src protein levels remained unaltered. Expression of Hsp90(**α** and **β**) was induced prominently in the proximal convoluted tubules (PCTs). Activation of Hsp90**α** was observed in vascular and outer medulla regions, whereas Hsp90**β** was induced in the cortex. Immunoreactivity of pc-Src was elevated in PCT with obvious staining at the luminal membrane. This *in vivo* study is the first to demonstrate that aldosterone nongenomically elevates Hsp90(**α** and **β**) protein expressions in rat kidney. Aldosterone had no effect on pc-Src protein levels but modulated localization. These results indicate that aldosterone regulates upstream mediators of EGFR transactivation *in vivo*.

## 1. Introduction

Aldosterone plays a major role in the maintenance of electrolytes and acid-base balances [1]. Besides genomic action, its nongenomic effects have been extensively investigated in various organs [2]. Previous *in vitro* studies indicated that nongenomic action of aldosterone is involved in transactivation of epidermal growth factor receptor (EGFR) and increases its downstream signaling kinases and extracellular signal-regulated kinases 1/2 (ERK1/2) [2–4]. A recent *in vivo* study was conducted in our laboratory. It was the first to demonstrate that aldosterone, via nongenomic pathway, elevated phosphorylated EGFR (pEGFR) and pERK1/2 protein abundances and expressions in rat kidney [5].

However, upstream signals induced by aldosterone consequently transactivating EGFR are still inconclusive. In *in vitro* studies, heat shock protein 90 (Hsp90) family and cytosolic tyrosine kinase of Src (c-Src) play a critical role in the initiating step of rapid nongenomic stimulation on EGFR [3, 6, 7]. Hsp90 family (*α* and *β*) is found in mineralocorticoid receptor (MR) complexes and is released upon aldosterone binding [8]. After dissociation, Hsp90*β* plays an influential role in signal transduction of nongenomic action by aldosterone [6, 7]. c-Src, a member of membrane-associated nonreceptor tyrosine kinases, has multiple biological functions, including a role in EGFR phosphorylation [9, 10].

In M-1 cells, aldosterone enhanced protein abundance of Hsp90*β* [6]. It increased activity as well as autophosphorylation of Src kinase and protein expression of pc-Src [6]. Moreover, stabilization of Hsp90*β* could prevent EGFR transactivation, while inhibition of c-Src kinase abolished aldosterone action in enhancement of pEGFR [7]. To date, there are no studies regarding nongenomic action of aldosterone on protein expression of Hsp90*α*.

At present, there are no available *in vivo* data regarding nongenomic effects of aldosterone on protein abundance and localization of renal Hsp90(*α* and *β*) and pc-Src, simultaneously performed in the same study. Therefore, this study examined rat kidneys 30 minutes after normal saline solution or aldosterone injection with use of Western blot analysis and immunohistochemistry to determine protein abundance and localization of renal Hsp90(*α* and *β*) and pc-Src. 

## 2. Materials and Methods

### 2.1. Experimental Design

 Male Wistar rats weighing 200–240 g (National Center of Scientific Use of Animals, Mahidol University, Nakornpathom, Thailand) were given conventional housing and diet. All animal protocols were approved by the Ethics Committee of Research, Chulalongkorn University. Serum creatinine of each rat should be <1 mg/dL [5]. The rats were divided into two groups (*n* = 8/group): sham (normal saline solution; NSS: 0.5 mL/kg BW by intraperitoneal injection, i.p.); and Aldo (aldosterone 150 *μ*g/kg BW, diluted in NSS, i.p.; Sigma, St. Louis, MO, USA) [5].

On the experimental date, after a 30-minute injection period of NSS or aldosterone, the rats were anesthetized with thiopental (100 mg/kg BW, i.p.). Kidneys were removed, and a half of each kidney was fixed in liquid nitrogen and then stored at −80°C until use for measurement of Hsp90*α*, Hsp90*β*, and pc-Src protein abundances by Western blot analysis. The other half of renal tissue was fixed in 10% paraformaldehyde for localization of these proteins by immunohistochemistry [5].

### 2.2. Western Blot Analysis

 The measurement of protein abundance was performed as previously described [5, 11]. Proteins were resolved on 10% sodium dodecyl sulfate polyacrylamide gel eletrophoresis (SDS-PAGE) for Hsp90*α*, Hsp90*β*, or pc-Src and blotted onto nitrocellulose membrane (Bio-Rad, Hercules, CA, USA). Membranes were incubated with primary monoclonal antibody to Hsp90*α* (D7a; 1 : 500), Hsp90*β* (H90-10; 1 : 1000) (Abcam, Cambridge, UK) [12], polyclonal antibody to pc-Src (Tyr418; 1 : 500) (MBL, Woburn, MA, USA), or to *β*-actin (Santa Cruz Biotechnology, Santa Cruz, CA, USA), followed by the respective horseradish peroxidase-linked secondary antibody (Bio-Rad). Immunoreactive proteins were detected by chemiluminescence detection (SuperSignal West Pico kit; Pierce, Rockford, IL, USA) and exposed to film (CL-XPosure; Pierce). Relative protein levels of Hsp90*α*, Hsp90*β*, or pc-Src in each sample were presented as a percentage of the control normalized to its *β*-actin content. 

### 2.3. Immunohistochemical Study

Detection of protein localization was performed as previously described [5]. Paraffin-embedded kidney sections were cut at 4 *μ*m in thickness. Slides were deparaffinized, and endogenous peroxidase was blocked by treatment with 3%  H_2_O_2_. Sections were incubated with the primary antibody Hsp90*α* (1 : 100), Hsp90*β* (1 : 200) (Abcam), or pc-Src (1 : 400) (MBL) at 4°C overnight, followed by the respective horseradish peroxidase-linked secondary antibody (Bio-Rad), and then reacted with 3, 3′-diaminobenzidine (DAB) solution (Sigma). Three pathologists independently scored staining intensity on a semiquantitative five-tiered grading scale from 0 to 4 (0 = negative; 1 = trace; 2 = weak; 3 = moderate; 4 = strong) as previously described [5].

### 2.4. Statistical Analyses

 Results of renal Hsp90*α*, Hsp90*β*, or pc-Src protein abundances were expressed as mean ± SD. Statistical differences among groups were assessed by ANOVA (analysis of variance) with post hoc comparison by Tukey's test where appropriate. A *P* value of <0.05 was considered statistically significant. Statistical tests were analyzed using SPSS program version 15.0 (SPSS Inc, Chicago, IL, USA). Median staining intensity (score) of renal Hsp90*α*, Hsp90*β*, or pc-Src protein expressions was presented as previously described [5].

## 3. Results

### 3.1. Effect of Aldosterone on Renal Hsp90*α*, Hsp90*β*, and pc-Src Protein Abundances

**Figure 1:**
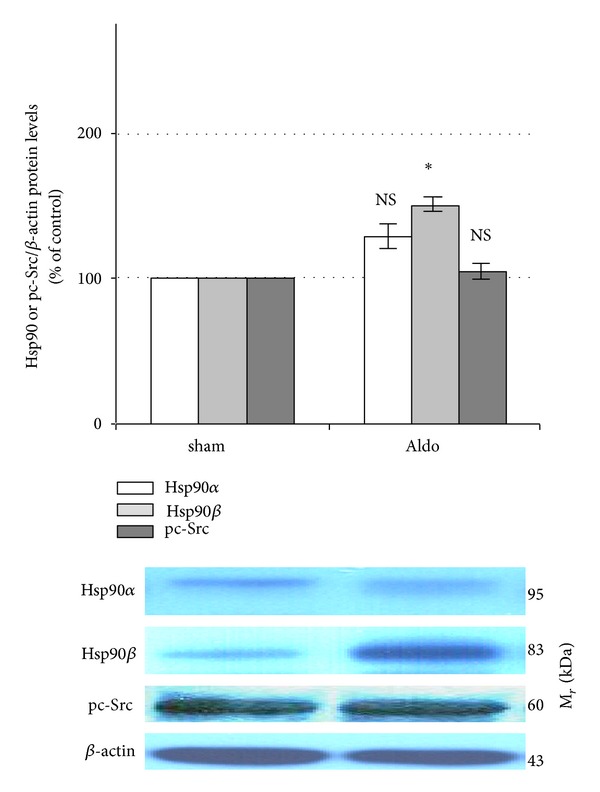
Western blot analysis of renal Hsp90*α*, Hsp90*β*, and pc-Src protein abundances in sham and Aldo groups. Histogram bars show the densitometric analyses ratios of Hsp90*α*, Hsp90*β*, or pc-Src to *β*-actin intensity, and the representative immunoblot photographs are presented. Data are means ± SD of 8 independent experiments. *P < 0.001 compared with the sham group.

Protein levels of Hsp90*α* (95 kDa), Hsp90*β* (83 kDa), and pc-Src (60 kDa) were assessed in rat kidney with Western blot analysis. As shown in Figure 1, aldosterone slightly enhanced protein abundance of renal Hsp90*α* (sham = 100%; Aldo = 128.5 ± 15.1%, *P* = 0.07), whereas protein level of Hsp90*β* was significantly increased to be 149.8 ± 9.2% (*P* < 0.001). However, protein level of renal pc-Src was unaltered in the Aldo group (*P* = 0.26). 

### 3.2. Effect of Aldosterone on Renal Hsp90*α* Protein Localization

**Figure 2:**
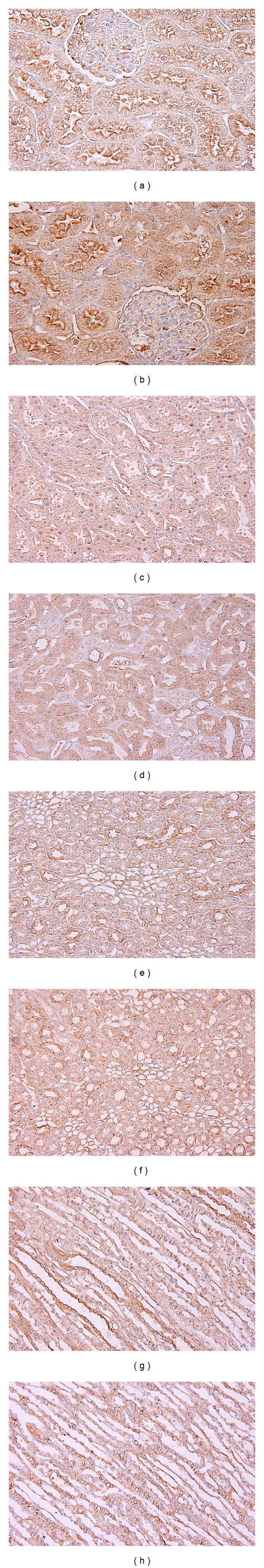
Representative immunohistochemical staining micrographs of renal Hsp90*α* protein expression in the cortex (a), (b), the outer medulla (outer stripe: (c), (d)), (inner stripe: (e), (f)) and the inner medulla ((g), (h)) from sham ((a), (c), (e), (g)) and Aldo ((b), (d), (f), (h)). Original magnification, ×400 ((a), (b)) and ×200 ((c)–(h)).

Rapid action of aldosterone on Hsp90*α* expression in rat kidney was examined by using immunohistochemistry. As shown in Table 1, in the cortex of sham, immunoreactivity of renal Hsp90*α* protein distribution and localization was diffused in both vascular and tubular regions with more intense staining at the luminal membrane (Figure 2(a)). Aldosterone increased intensity score in the proximal convoluted tubule (PCT) from 2 to be 3 and in peritubular capillary (Pcap) from 1 to be 2 (Figure 2(b)). Immunoreactivity in the CCD was slightly diminished to be 2 by aldosterone.

In the outer stripe of outer medulla (OM) of the Aldo group, immunoreactivity in the thick ascending limb of Henle's loop (TALH) and medullary collecting duct (MCD) remained (Figures 2(c) and 2(d)), whereas staining in the proximal straight tubule (PTs) was diminished to be 1. In the inner stripe of OM (Figures 2(e) and 2(f)), aldosterone increased staining in the TALH (score = 4), vasa recta (VR; score = 3), and thin limb of Henle's loop (tLH; score = 2). In the inner medulla (IM), immunoreactivity was elevated in the VR, whereas the expression in the MCD was reduced by aldosterone (Figures 2(g) and 2(h); Table 1).

### 3.3. Effect of Aldosterone on Renal Hsp90*β* Protein Localization

**Figure 3:**
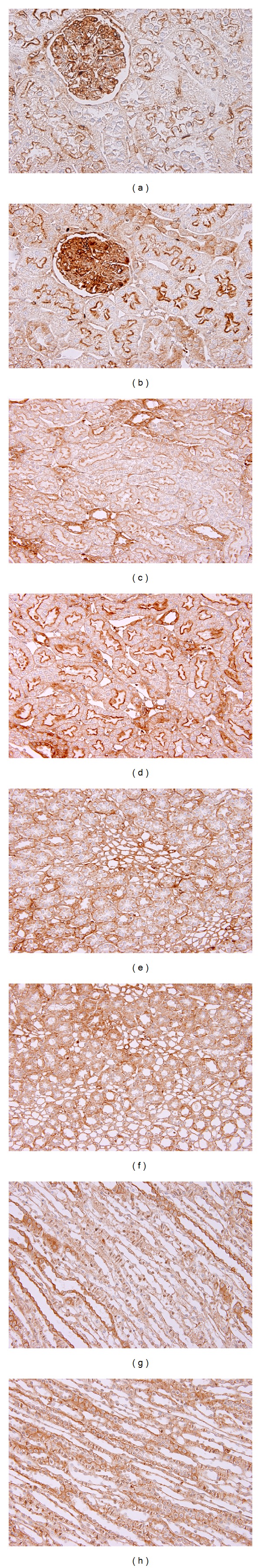
Representative immunohistochemical staining micrographs of renal Hsp90*β* protein expression in the cortex ((a), (b)), the outer medulla (outer stripe: (c), (d)), (inner stripe: (e), (f)) and the inner medulla ((g), (h)) from sham ((a), (c), (e), (g)) and Aldo ((b), (d), (f), (h)). Original magnification, ×400 ((a), (b)) and ×200 ((c)–(h)).

 Protein expression of Hsp90*β* in the cortex of sham was demonstrated in Figure 3(a) and Table 1. Immunoreactivity was moderate at the glomerulus and Pcap. Prominent staining in the luminal membrane of PCT was noted, while expression in DCT and CCD was trace. Aldosterone stimulated strong immunoreactivity in the glomerulus, whereas staining in PCT and CCD was moderate (Figure 3(b)). The Aldo group showed weak staining in the DCT and intensity was diminished in the Pcap. 

In the outer stripe of OM, aldosterone did not alter strong immunoreactivity in the TALH and MCD (Figures 3(c) and 3(d)) but increased the intensity score in the PTs to be 3. In the inner stripe of OM, aldosterone did not change immunoreactivity in the TALH, MCD, and tLH, whereas staining in VR was diminished (Figures 3(e) and 3(f); Table 1). In the IM, immunoreactivity was enhanced in tLH, while intensity score was reduced in MCD (Figures 3(g) and 3(h)). 

### 3.4. Effect of Aldosterone on Renal pc-Src Protein Localization

**Figure 4:**
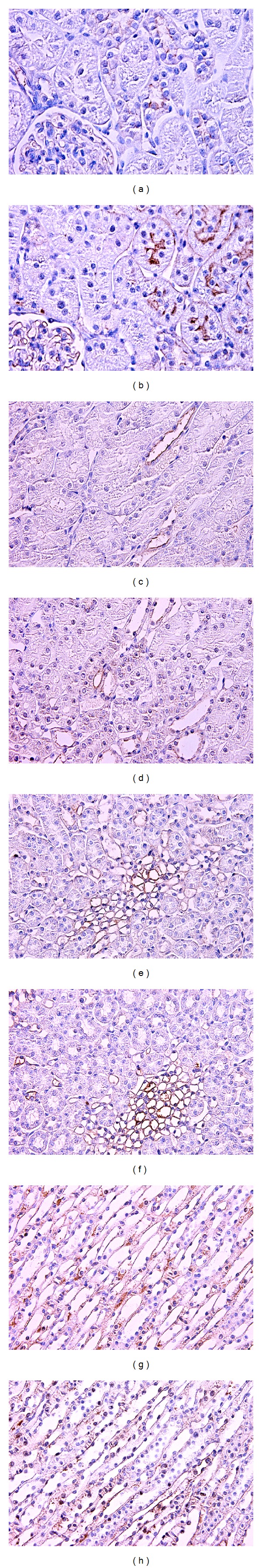
Representative immunohistochemical staining micrographs of renal pc-Src protein expression in the cortex ((a), (b)), the outer medulla (outer stripe: (c), (d)), (inner stripe: (e), (f)) and the inner medulla ((g), (h)) from sham ((a), (c), (e), (g)) and Aldo ((b), (d), (f), (h)). Original magnification, ×400 ((a), (b)) and ×200 ((c)–(h)).

In the cortex of sham, immunoreactivity of pc-Src protein was trace in the glomerulus and DCT, whereas weak staining at the CCD luminal membrane was noted (Figure 4(a); Table 1). In the sham group, we observed no staining in the PCT and Pcap. Aldosterone markedly enhanced immunoreactivity in the PCT (score = 3) with more intense staining at the luminal membrane and lesser extent at the basolateral membrane (Figure 4(b)), whereas staining in the glomerulus was moderate. Immunoreactivity was diminished in the DCT and CCD by aldosterone. 

In the outer stripe of OM, aldosterone slightly reduced immunoreactivity in TALH and MCD to be trace-diffuse staining (Figures 4(c) and 4(d)). Aldosterone had no action on protein expression in PTs. In the inner stripe of OM, aldosterone enhanced the immunoreactivity in VR and tLH (Figures 4(e) and 4(f)) but staining disappeared in TALH and MCD. In the IM, aldosterone diminished the MCD intensity score leaving VR and tLH staining unaltered (Figures 4(g) and 4(h)). 

## 4. Discussion

Our recent *in vivo* study has demonstrated that aldosterone, via nongenomic pathway, could transactivate EGFR and enhance a downstream-signal ERK1/2 protein abundance and expression in the rat kidney [5]. Previous *in vitro* examinations indicated that Hsp90*β* and c-Src are the upstream mediators transactivating EGFR [3, 6, 7]. There are no *in vivo* data of these aldosterone-induced rapid action mediators. The present investigation aims to examine the protein expressions of these upstream signals in the rat kidney.

Here we present the first *in vivo* results that simultaneously showed both renal Hsp90*α* and Hsp90*β* protein expressions after 30-minute aldosterone administration. Protein abundance of renal Hsp90*β* was significantly enhanced but protein level of Hsp90*α* remained unaltered. This result of Hsp90*β* is similar to a previous study in M-1 cell culture examined [6]. Baseline regional localization and distribution of these proteins in this study are in agreement with an earlier investigation in normal rat kidney [12]. Aldosterone induced the expression of Hsp90(*α* and *β*) prominently in the PCT. Greater activation of Hsp90*α* was observed in vascular areas and outer medulla regions, whereas Hsp90*β* was induced mainly in the cortex. 

In addition, as an upstream signal for EGFR transactivation, Hsp90 can itself regulate cellular functions. In microdissected CCD, Hsp90 stimulated calcineurin activity within 15 minutes [13]. A 20-minute incubation period of Hsp90 was found to stabilize Na,K-ATPase in cytoskeletal fractions of ischemic rat renal cortex [14]. Geldanamycin, an inhibitor of Hsp90, abolished aldosterone-induced vasorelaxation of renal afferent arterioles within 20 minutes through nitric oxide generation [15]. Moreover, Hsp90*β* induced by aldosterone could rapidly elevate Src kinase activity [6]. It has been shown that Hsp90*α* transfected in human embryonic kidney cells induced endothelial nitric oxide synthase ability to produce nitric oxide, while Hsp90*β*-transfected cells generated superoxide anion [16]. However, there are no data of these alterations occurring *in vivo* via nongenomic action of aldosterone. Further investigation is needed to clarify this point. 

Another upstream signal, cytosolic nonreceptor tyrosine kinase, c-Src, has been demonstrated to link aldosterone-bound MR and EGFR activation [6, 7, 17–20]. Aldosterone was shown to rapidly enhance c-Src kinase activity and transactivated EGFR [6, 7, 17–20]. An increased EGFR phosphorylation mediated by aldosterone/c-Src induction was inhibited by the c-Src inhibitor, PP2 [17–20]. In M-1 cell, aldosterone rapidly induced pc-Src protein levels in a dose-dependent manner [6]. Furthermore, this protein abundance in vascular smooth muscle cells was time dependently increased by aldosterone (from 15 to 45 minutes) [17, 18]. However, there are no data so far to pc-Src protein abundance and localization/distribution in rat kidney by rapid action of aldosterone. 

 Present results of this study show that pc-Src protein abundance was not altered by aldosterone (Figure 1). It is plausible that protein degradation occurred thus returning to the basal level as a result of the 30-minute time period after which aldosterone was administered. Aldosterone in M-1 cells was also shown to undergo a time-dependent induction in c-Src kinase activity at the 2nd and 10th minute [6]. This activity was comparable to the control at the 30th minute. To better understand protein abundance performed at the shorter time length, further *in vivo* studies are needed.

As for pc-Src protein localization, the sham rat showed immunostaining in the glomerulus, VR, DCT, CD, and loop of Henle with no expression in PCT and PTs (Figure 4(a)). *In vivo* regional localization and distribution of active Src kinase in tubular areas offered some similarity to those of previous studies [19, 20]. In this particular study though, aldosterone markedly enhanced immunostaining in PCT with prominent luminal membrane expression (Figure 4(b)). Explanation of the precise mechanism in this observation remains unclear. However, these important data present strong supportive evidence that, in addition to its action as an upstream signal, c-Src might play another crucial role in proximal tubular functions as well. An example of this dual role is demonstrated through the enhancement of c-Src kinase activity in OKP cells within 1.5 minute after acid loading [21]. This stimulation of c-Src kinase is required for acid-induced sodium/hydrogen exchanger-3 (NHE-3) activation. Moreover, acid incubation in OKP cells expressing dominant negative *c*-*src* ^*K*295*M*^  caused no effect on NHE-3 activity [21]. 

Our study has shown aldosterone to enhance pc-Src expression in the glomerular region and VR area (Figures 4(b), 4(f), and 4(h)). This may indicate c-Src regulation of the glomerular function and renal microvasculature. An overexpression of c-Src in murine mesangial cells has been demonstrated to augment NF-*κ*B activation and nitric oxide production [22]. Furthermore, c-Src activation by *in vitro* blood-perfused juxtamedullary nephron technique contributed to afferent arteriole constriction induced by angiotensin II [23]. We propose that c-Src may influence vasoactivity and consequently regulates blood circulation in the renal microvasculature; however, since no data of c-Src on VR responses has yet to be established, additional study is required to fully assess this issue. 

Indeed, several investigations have revealed that EGFR phosphorylation occurred after Hsp90 release from MR, thus leading to c-Src kinase activation [6, 7, 17–20]. Earlier studies have been successful at demonstrating that Hsp90, by itself, could also bind directly to EGFR thereby stabilizing receptor conformation [24, 25]. Moreover, besides the consequent activation by aldosterone, both Hsp90 and c-Src have a profound interaction. It has been illustrated that Hsp90 is necessary for the maturation of the tyrosine kinase c-Src as a kinase and as a substrate in yeast cells [26], yet there are still no available data relating to this regard in other species. Therefore, interaction of aldosterone/Hsp90/c-Src/EGFR is likely complex. The complexity of this interaction warrants further *in vivo* examinations. Additional investigations using the blockage of Hsp90(*α* and *β*) or c-Src or MR are also needed.

## 5. Conclusions

 This is the first *in vivo* study which demonstrates that aldosterone, via nongenomic pathway, could enhance Hsp90(*α* and *β*) protein expressions in rat kidney. Although aldosterone was not found to alter pc-Src protein levels, its localization was modulated. Our data indicate that aldosterone regulates upstream mediators of EGFR transactivation *in vivo.*

## Figures and Tables

**Table 1:** Median staining intensity (score) of renal Hsp90*α*, Hsp90*β*, and pc-Src protein expressions.

	Median staining intensity (score)					
	Hsp90*α*		Hsp90*β*		pc-Src	
	sham	Aldo	sham	Aldo	sham	Aldo
Cortex						
Glomerulus	2	2	3	4	1	2
PCT	2	3	2	3	0	3
DCT	1	1	1	2	1	0
CCD	3	2	1	3	2	1
Pcap	1	2	3	1	0	0

Outer medulla						
Outer stripe						
TALH	3	3	4	4	2	1
MCD	2	2	4	4	2	1
PTs	2	1	2	3	0	0
Inner stripe						
TALH	3	4	3	3	1	0
MCD	3	3	3	3	1	0
VR	2	3	4	3	3	4
tLH	1	2	2	2	1	4

Inner medulla						
MCD	4	3	4	3	2	1
VR	2	3	2	2	4	4
tLH	2	2	2	3	2	2

Staining intensity: 0: negative, no reactivity; 1: trace, faint, or pale brown staining with less membrane reactivity; 2: weak, light brown staining with incomplete membrane reactivity; 3: moderate, shaded of brown staining of intermediate darkness with usually almost complete membrane reactivity; 4: strong, dark brown to black staining with usually complete membrane pattern, producing a thick outline of the cell [5].

PCT: proximal convoluted tubule; DCT: distal convoluted tubule; CCD: cortical collecting duct; Pcap: peritubular capillary; TALH: thick ascending limb of Henle's loop; MCD: medullary collecting duct; PTs: proximal straight tubule; VR: vasa recta; tLH: thin limb of Henle's loop.
